# The cross-correlation-based analysis to digest the conformational dynamics of the mitoBK channels in terms of their modulation by flavonoids

**DOI:** 10.1007/s00249-023-01666-9

**Published:** 2023-06-30

**Authors:** Agata Wawrzkiewicz-Jałowiecka, Paulina Trybek, Beata Dworakowska, Piotr Bednarczyk, Przemysław Borys

**Affiliations:** 1 Department of Physical Chemistry and Technology of Polymers, Silesian University of Technology, Strzody 9, Gliwice, 44-100 Poland; 2 Institute of Physics, University of Silesia in Katowice, 75 Pułku Piechoty 1A, Chorzów, 41-500 Poland; 3 Institute of Biology, Department of Physics and Biophysics, Warsaw University of Life Sciences - SGGW, Nowoursynowska 159, Warsaw, 02-787 Poland

**Keywords:** MitoBK channels, Cross-correlations, Conformational dynamics, Flavonoids, Channel gating dynamics, Dwell-time series

## Abstract

The activity of mitochondrial large-conductance voltage- and $Ca^{2+}$-activated $K^+$ channels (mitoBK) is regulated by a number of biochemical factors, including flavonoids. In particular, naringenin (Nar) and quercetin (Que) reached reasonable scientific attention due to their well-pronounced channel-activating effects. The open-reinforcing outcomes of Nar and Que on the mitoBK channel gating have been already reported. Nevertheless, the molecular picture of the corresponding channel–ligand interactions remains still to be revealed. In this work, we investigate the effects of the Nar and Que on the conformational dynamics of the mitoBK channel. In this aim, the cross-correlation-based analysis of the single-channel signals recorded by the patch-clamp method is performed. The obtained results in the form of phase space diagrams enable us to visually monitor the effects exerted by the considered flavonoids at the level of temporal characteristics of repetitive sequences of channel conformations. It turns out that the mitoBK channel activation by naringenin and quercetin does not lead to the change in the number of clusters within the phase space diagrams, which can be related to the constant number of available channel macroconformations regardless of the flavonoid administration. The localization and occupancy of the clusters of cross-correlated sequences suggest that mitoBK channel stimulation by flavonoids affects the relative stability of channel conformations and the kinetics of switching between them. For most clusters, greater net effects are observed in terms of quercetin administration in comparison with naringenin. It indicates stronger channel interaction with Que than Nar.

## Introduction

Large-conductance voltage- and Ca$^{2+}$-activated $K^+$ channels (BK) play important and specific roles in a plethora of physiological processes, which affect the functioning of both healthy and pathological cells (Latorre et al. 2017; Contreras et al. 2013). The BK channels are encoded by the KCNMA1 gene (Singh et al. 2013). They are located not only in the plasma membrane but also in the inner mitochondrial membrane (Balderas et al. 2015).

The possibilities of chemical modulation of the mitochondrial BK channels (mitoBK) received a scientific interest in recent years due to the involvement of these channels in regulation of metabolism and cytoprotective processes (Kulawiak et al. 2008; Szabo and Zoratti 2014; Krabbendam et al. 2018; Wawrzkiewicz-Jałowiecka et al. 2023). Among the reported substances that increase the transport capabilities of the mitoBK channel, one can mention NS1619 (Xu et al. 2002), NS11021 (Kicinska et al. 2020), CGS7184 and CGS7181 (Augustynek et al. 2018) and two natural flavonoids, i.e., naringenin (Nar) (Testai et al. 2017; Kampa et al. 2019; Kicinska et al. 2020) and quercetin (Que) (Kampa et al. 2021, 2022). Owing to the fact that the most problems related to the modulating mitoBK channels for therapeutic purposes are associated with the possible wide spectrum of off-target effects (Wrzosek et al. 2020), the substances listed last deserve a particular attention.

**Fig. 1:**
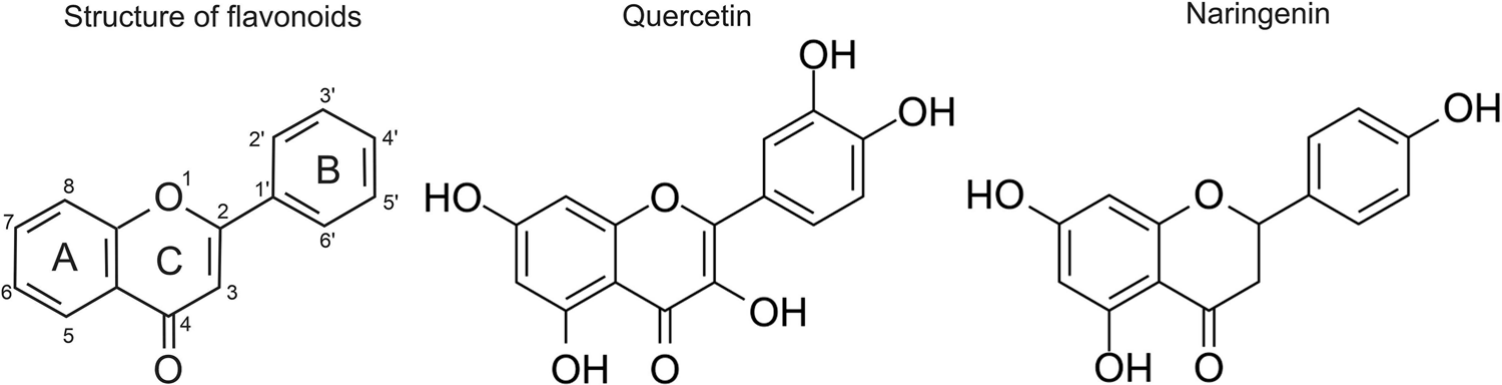
Chemical structures of the flavonoids, including the general structure (left), quercetin (middle) and naringenin (right)

Flavonoids are a diverse group of plant metabolites, widely distributed in various fruits, vegetables, grains, herbs, and beverages such as tea and wine. They are characterized by their chemical structure, which includes a 15-carbon skeleton with 2 aromatic rings (A and B) joined by a 3-carbon bridge (C) (see Fig. 1) (Kumar and Pandey 2013; Wen et al. 2017). Recently, quercetin (PubChem CID: 5280343) and naringenin (PubChem CID: 932) have gained reasonable scientific attention due to their pronounced channel-activating effects (Testai et al. 2017; Kampa et al. 2019; Kicinska et al. 2020; Kampa et al. 2021, 2022; Richter-Laskowska et al. 2023). Quercetin, 2-(3,4-dihydroxyphenyl)-3,5,7-trihydroxychromen-4-one, is a flavonoid commonly found in various fruits, vegetables, and grains. It has been reported that exposure to light (particularly ultraviolet light) can lead to the degradation of quercetin molecules, resulting in reduced stability and potential loss of its biological activity (Golonka et al. 2020). Another flavonoid primarily found in citrus fruits such as oranges and grapefruits is naringenin (5,7-dihydroxy-2-(4-hydroxyphenyl)-2,3-dihydrochromen-4-one). Like quercetin, naringenin can also be sensitive to light, especially ultraviolet light. When exposed to light, naringenin may undergo photochemical reactions that can alter its chemical structure and potentially affect its properties and biological activity (Ioannou et al. 2018).

Naringenin and quercetin are representatives of flavonoids being considered as natural nutraceuticals due to their anti-oxidative, anti-inflammatory, anti-mutagenic and anti-carcinogenic properties (Ullah et al. 2020). For the sake of the beneficial effects for health, their pharmacological utilization as mitoBK channel activators are largely devoid of serious side effects. The basic description of the biological consequences of the mitoBK channel modulation by Nar and Que have been already provided in the literature both at the cellular level (cytoprotection) and the molecular one (i.e., in terms of the elementary characteristics of single-channel kinetics) (Testai et al. 2017; Kampa et al. 2019; Kicinska et al. 2020; Kampa et al. 2021, 2022; Richter-Laskowska et al. 2023). Nevertheless, the molecular mechanisms of how these flavonoids can affect the conformational dynamics of the mitoBK channel are still largely unknown.

The current knowledge indicates that the mitochondrial BK channel structure is a kind of reflection of the plasma membrane structure (Balderas et al. 2015). Therefore, the BK and mitoBK channel $\alpha$ subunits have many common structural characteristics and, frequently, the cryo-EM structures of the BK channel (PDB ID 6V22 and 6V3G) (Tao and MacKinnon 2019) can serve as rational templates to investigate the main structure–function relationships for both channel variants. Analogously, the popular kinetic models of the BK channel gating are still applicable to the mitoBK channels. Nevertheless, one should remember that some minor structural differences between the mitochondrial and plasma membrane BK channel isoforms may occur due to the alternative splicing during the Kcnma1 gene transcription or some post-translational modifications.

**Fig. 2:**
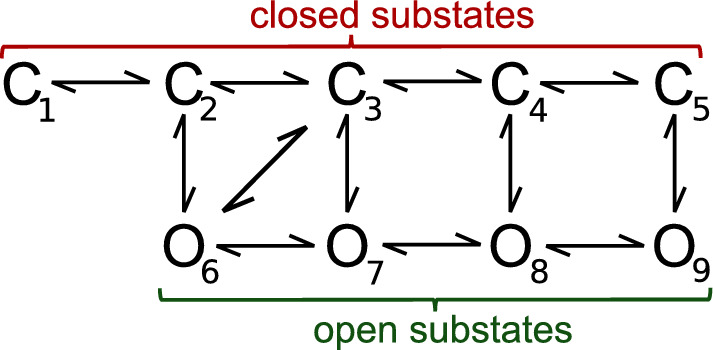
Exemplary kinetic scheme representing channel gating. The substates $O_i$ and $C_i$ denote the possible open and closed conformations, respectively. The arrows stand for the possible transitions between the channel substates

The most popular molecular picture illustrating the random open–closed (i.e., conducting–non-conducting) fluctuations of the channel structure assumes the existence of several discernible conformations. In the case of the BK-type channels, this can be seen by the molecular dynamics (MD) studies (Tao and MacKinnon 2019). The number of the BK channel conformations remains not affected, e.g., by the interactions with the regulatory $\beta$ subunits (Tao and MacKinnon 2019). In the context of modeling of the BK channel activity in a relatively long time scale (exceeding the MD limits), frequently a multistate Markovian model is used (Geng and Magleby 2015), as schematically presented in Fig. 2. At fixed Ca$^{2+}$ concentration and constant membrane potential ($U_m$), the kinetic scheme of 3–4 open and 5–6 closed substates representing stable channel conformations can describe the BK channel gating well (Geng and Magleby 2015).

It turns out, however, that the exact number of the substates corresponding to the stable BK channel conformations, their dwell-time characteristics as well as the system of their interconnections are not known a priori. From this perspective, to extract some practical information about the gating dynamics, advanced computational methods should be used.

In this paper, we would like to characterize the mitoBK channel gating in terms of its activation by naringenin and quercetin to make progress in our understanding of the channel modulator interactions, and its functional consequences for the conformational dynamics of the channel protein. We would like to see whether these two drugs affect the mechanism of switching between the channel’s conformations in a similar way or not. We also aim to investigate the strength of the channel modulator interactions not only at the “global” level of the kinetics of channel’s open and closed macroconformations (as usually, by evaluation of open state probability, mean open and closed dwell-times), but rather refer to the kinetics of the channel conformations. This kind of research seems to be valuable from the perspective of the possible extraction of key features of modulators that can act in the even more effective and specific way on the mitoBK channels, as well as regulate its functioning in an intentional and controlled manner.

Inspecting the exemplary kinetic diagram in Fig. 2, one can expect that sufficiently long observation of the system’s behavior should allow for the detection of all possible transitions between channel substates. The more energetically favorable channel substates should be occupied more frequently than the high-energetic ones. The acts of switching, which are described by larger rate constants, should occur more frequently than the ones of small rate constants. These regularities predetermine pattern formation in the temporal characteristics of the subsequent channel substates which is a starting point for our investigations. The analysis of sufficiently long recordings of single-channel currents allows for the construction of the corresponding series of dwell-times of the successive open and closed states of the channel, which by the reasoning provided above, should exhibit some signs of repeatability. This kind of repeatability should, in turn, enable making inferences about the underlying conformational dynamics, which was already postulated in Labarca et al. (1985); Colquhoun and Hawkes (1987, 1995). In those studies, the authors tried to estimate the number of entry routes to functional states of ion channels based on the investigation of the existing correlations between successive dwell-times of open and closed states.

According to that idea, we will analyze the correlations and repetitivity of dwell-time sequences. In particular, we will utilize the methodology recently presented in Borys et al. (2022) and visually monitor the effects induced by flavonoid binding on conformational dynamics of the mitoBK channels by use of three-dimensional phase diagrams in “dual space”. These diagrams represent the sets of correlated sequences of subsequent dwell-times of the open-closed-open (O–C–O) and closed-open-closed (C–O–C) types that describe the discernible acts of switching between existing substates of the channel. Identification of the most occupied sets of the correlated O–C–O and C–O–C sequences and tracking the changes in their cardinality and location in phase space with the increase of Nar and Que concentrations can be considered as a unique reflection of the changes in conformational dynamics of a channel protein resulting from the channel–flavonoid interaction.

It is the first work where the nonlinear methods are applied to evaluate the effects exerted by different ligands on the conformational dynamics of the mitoBK channel. The presented cross-correlation-based analysis seems to be an approach that may be extensively exploited in ion channel research. This methodology is relatively fast and simple. It enables for inferences about the main characteristics of gating machinery in a long observation scale (the statistics of thousands acts of switching between channel states). In this aspect, our approach largely exceeds the current possibilities of the other modern computational methods, like molecular dynamics, at the price of a decrease in the description accuracy.

## Methods

### Cell culture

In this study, human endothelial cell line EA.hy926 was used. Cells were cultured under standard incubation conditions at 37 $^{\circ }$C and in a humidified atmosphere at 5% CO$_2$. The culture medium contained the following substances: Dulbecco’s modified Eagle’s medium (1 g/L D-glucose) enriched with 10% fetal bovine serum (FBS), 2% hypoxanthine–aminopterin–thymidine (HAT), 1% penicillin/streptomycin and 1% L-glutamine. Before the electrophysiological measurements, cells were cultured in 150 mm dishes for 6 days. It allowed for reaching approximately 90% of the maximum number of cells in the dish. Cells were passaged every third day. For the study, we used the cells derived between 5 and 20 passages.

### Mitochondria and mitoplast preparation

The detailed description of the procedures of mitochondria and mitoplast preparation for the electrophysiological measurements are provided in Bednarczyk et al. (2013) and Kicinska et al. (2020). In short, the mitochondria were isolated by series of differential centrifugation at 4$^{\circ }$C. First, the EA.hy926 cells were collected in PBS medium and centrifuged at 800 g for 10 min. Second, they were resuspended in preparation solution (250 mM sucrose and 5 mM HEPES; pH 7.2) and homogenized using the glass-glass homogenizer (no. 19, Kontes Glass). Then, in aim to dispose of the vesicles, the resulting homogenate was centrifuged at 9200 g for 10 min. To separate the fraction of purified mitochondria, the pellet was once more resuspended in preparation solution and centrifuged (790 g, 10 min). At last, two faster centrifugations were performed to remove the preparation solution (at 9200 g for 10 min).

The mitoplasts were obtained from the mitochondria by swelling induced by incubation in hypotonic solution containing 5 mM HEPES, 100 $\mu {\textrm{M}}$ CaCl$_2$ (pH 7.2) for ca. 1 min. After that, a hypertonic solution was added (750 mM KCl, 100 $\mu {\textrm{M}}$ CaCl$_2$, 30 mM HEPES; pH 7.2) to restore isotonicity of the medium.

### Electrophysiology

Single-channel mitoBK activity was recorded by the patch-clamp method in a mitoplast-attached mode. For control experiments, the symmetric solution was used in pipette and bath (10 mM HEPES, 150 mM KCl, and 100 $\mu$M CaCl$_2$; pH 7.2). The patch-clamp pipettes from borosilicate glass of a resistance of 10-20 M$\Omega$ were used. To stimulate the channel by flavonoids—naringenin and quercetin (Sigma Aldrich) were added as dilutions by the perfusion system to reach the 3 and 10 $\mu$M concentrations. The mitoplasts at the tip of the measuring pipette were transferred into the openings of a multi-barrel “sewer pipe” system, and their external faces were rinsed there with the test solutions.

The patch-clamp signals of ca. 20 s long duration were sampled at a frequency of 10 kHz by the operational amplifier Axopatch 200B (Molecular Devices Corporation, USA) and low-pass filtered at 1 kHz. The single-channel currents were recorded at $U_m$ = 40 mV (membrane depolarization). For each patch-clamp experiment, a fresh mitoplast was used. At each combination of flavonoid concentration, 3–7 independent recordings were obtained. The preliminary qualitative analysis of the obtained signals was carried out using Clampfit 10.7 software (Axon Instruments, Molecular Devices Corporation, USA).

In the course of the experimental work, it turned out that the quality of the investigated mitoplast patches notably worsened after multiple perfusion (i.e., control–Nar (3 $\mu$M)–Nar (10 $\mu$M)–washout–Que (3 $\mu$M)–Que (10 $\mu$M)). For this reason, we decided to perform separate and independent experiments with naringenin and quercetin stimulation. It significantly reduced the stages of perfusion twice and allowed us to obtain more reliable recordings. Due to this strategy, the results of further analysis are presented in two variants. The first variant, where the results obtained at naringenin and quercetin stimulation are compared with their ‘own’ controls. This kind of analysis allows one to evaluate the impact of flavonoids on the mitoBK gating dynamics. The second variant is when the results are presented and commented on in relation to the common control (mean).

The input data for the correlation-based analysis are dwell-time series of the subsequent open and closed channel states. They can be constructed from the original patch-clamp recordings just after finding the threshold value of the current which separates the open and shut states. In that aim, the procedure described by Mercik et al. (1999) was applied. The identification of the open and closed states for each value of recorded current allows for the evaluation of the cumulative sojourns in the subsequent states, which constitute the dwell-time series.

### Correlation-based algorithm

The full description of applied methodology is presented in Borys et al. (2022). Here, we summarize only the most important steps of analysis together with the main idea of the used algorithm. The clue of the implemented technique lies behind the identification of correlations between short sub-sequences of the dwell-time series. The correlation is defined here by a simple standard formula for the cross-correlation $R_{xy}$ between two sequences $X_i$ and $Y_i$ (1). It is given by the ratio of covariance to the root-mean variance:

1 $$\begin{aligned} R_{XY} = \frac{\sum _{t=1}^N [(X^t_i - \overline{X_i})(Y^t_i - \overline{Y_i})]}{\sqrt{\sum _{t=1}^N (X^t_i - \overline{X_i})^2 \sum _{t=1}^N (Y^t_i - \overline{Y_i})^2}} \end{aligned}$$

where N is the length of the considered subseries. Here, we assume $N=3$. It is an optimal choice taking into account the possibility for the visualization of the results (each element of a sequence can be put on a single axis). Moreover, the choice of $N=3$ increases the likelihood for obtaining the reliable statistical representation of the separate sequences, which correspond to the switching between different channel substates. Briefly, the algorithm comprises two main steps.

**Preliminary grouping of data**

1. First, the list of N-element long sequences of dwell-times is constructed with careful attention to start from either an open (O–C–O) or a closed (C–O–C) state, respectively. This list should be sorted according to the decreasing value of the product of the dwell-times constituting each sequence and indexed. The first *N*-element sequence represents a comparative series, and its index is excluded from the list.
2. The comparative series is correlated with the next non-excluded *N*-element sequence using a formula (1). If the cross-correlation value is higher or equal to the introduced threshold $R_0$, the weighted average of the considered subseries is calculated and taken as a new comparative series. The index of the newly added sequence is excluded.
3. Step 2 should be executed (taking the next non-excluded sequence) until all of the N-element long sequences of a particular type (O–C–O or C–O–C) have been compared to the comparative series.
4. From the remaining elements of the initial input series (i.e., the ones for which their indexes have not been excluded yet), one should execute the operations from steps 1 and 2. In this way, another set of cross-correlated sequences is generated.
5. Steps 2, 3, 4 should be repeated until the input data are exhausted.

**Optimization**

1. For each set of dwell-time sequences obtained after the initial data separation, one should check whether all sequences included are cross-correlated to the final comparative sequence ($\ge R_0$). If insufficiently correlated ones are found, they should be rejected from the current set and incorporated into another one for which they exhibit the sufficient cross-correlation ($\ge R_0$), or they should form a seed for another cluster. After rejecting/adding a given sequence from/into a set, the corresponding comparative sequence should be updated.
2. The preceding step should be repeated until no more corrections are needed and the optimal data separation is reached.

The calculations should be made for different values of the $R_0$ parameter. As the most appropriate threshold value, one should choose the highest one, which allows for obtaining single-exponential distributions of dwell-times within most or, ideally, all the sets. (The optimal $R_0$ was 0.75 in the current research.)

The whole procedure should be repeated for the sequences starting from another functional state (i.e., of the C–O–C type, if first only the O–C–O sequences were considered).

**Visualization**

The obtained sets of the cross-correlated, 3-element-long dwell-time sequences are represented by circles positioned in the average 3D dwell-time coordinates. Diameter of each circle is proportional to the relative cardinality (occupancy) of a given set.

As mentioned before, in Section Electrophysiology, the results are presented in Section Results and discussion in two variants—first, with individual controls for both Nar- and Que-activation and, second, with common control. It is a consequence of the fact that the control results alone display differences due to unknown environmental factors, which are impossible to be controlled in the experiment. To construct the clusters in the second variant, the common control (CC) was introduced as average from the Nar- and Que-controls. To keep the possibility to investigate effects of flavonoid administration in relation to the CC, the other clusters describing the quercetin-induced activation of the channel were translated by a vector being the distance of Que-related control to the common control. Analogously, the original Nar-related clusters were translated by a vector given as a distance of the Nar-related control to the common control.

**Analysis of dispersion**

To track the changes in dispersion of the sets of cross-correlated sequences in phase space with a given parameter (here, flavonoid concentration), we introduce an appropriate measure ($<dist_{\tau }>$). The $<dist_{\tau }>$ parameter is defined as the minimal length of a loop connecting all distinguished clusters of the cross-correlated sequences at fixed external conditions divided by a number of these clusters. The error of $<dist_{\tau }>$ estimation can be calculated basing on the individual errors of the clusters’ positions via the total differential method.

## Results and discussion

The single-channel recordings obtained in this research confirmed the channel-activating effects exerted by naringenin and quercetin, as presented in Table 1. Quercetin administration resulted in a higher increase of the open state probability in comparison to naringenin, which is in agreement with the literature (Kicinska et al. 2020; Kampa et al. 2021). In addition, the correlation analysis performed on these recordings revealed many new details.

**Table 1:** The open state probabilities ($p_{op}$s) for the mitoBK channels obtained for the experimental data at two concentrations of naringenin (Nar) and quercetin (Que), i.e., 3 and 10 $\mu$M, and the corresponding controls in terms of membrane depolarization ($U_m$ = 40 [mV])

	control	3 [$\mu$M]	10 [$\mu$M]
Que	0.58±0.03	0.80±0.04	0.85±0.03
Nar	0.56±0.05	0.63±0.02	0.66±0.01

The presented $p_{op}$s are given as the average values ± standard error

**Fig. 3:**
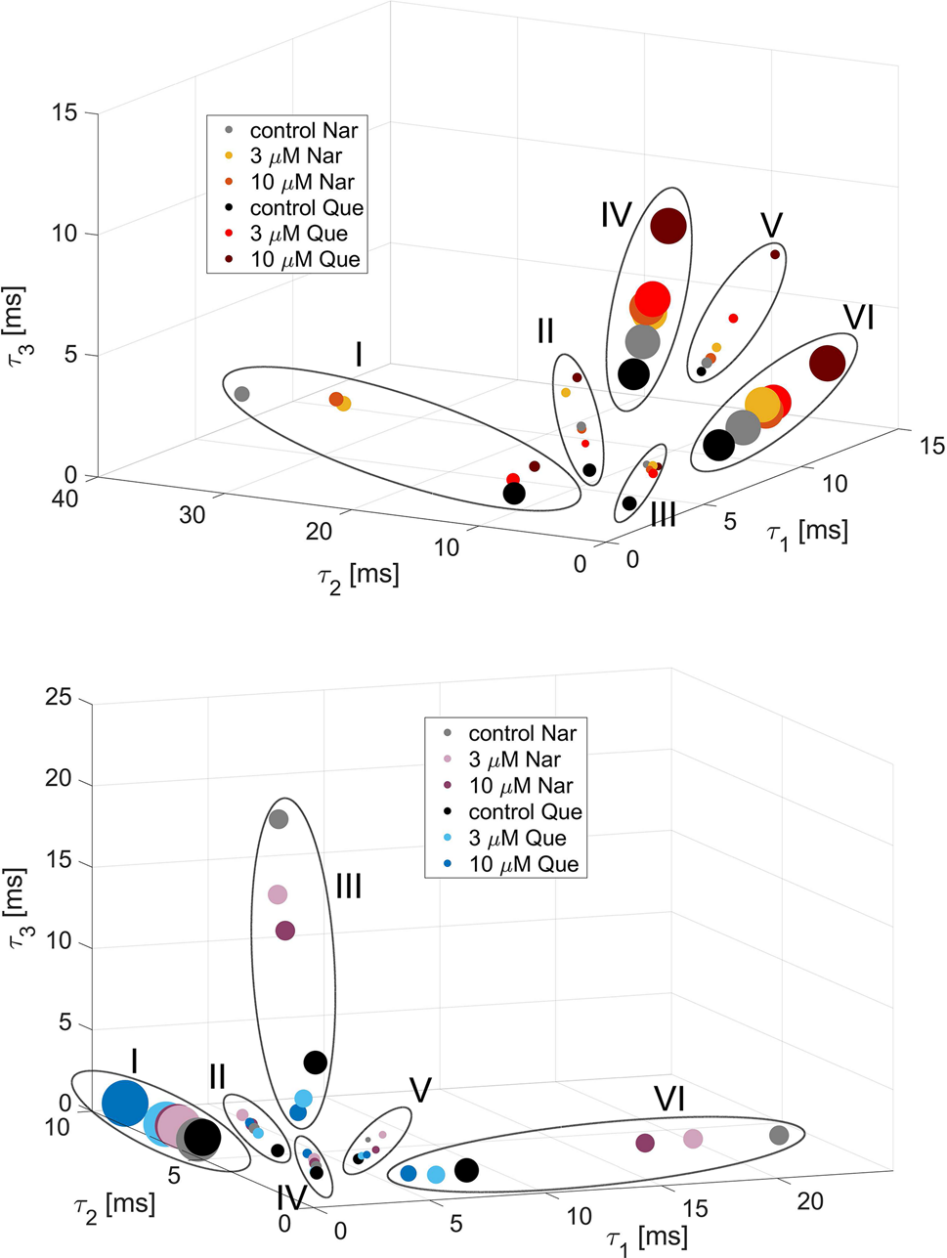
The phase space representation of the clusters of the cross-correlated O–C–O (top) and C–O–C (bottom) sequences at $R_0$ = 0.75 threshold, describing channel’s gating dynamics induced by the naringenin and quercetin binding, and their individual control groups. Each cluster is encircled by a dashed line and numbered. The coordinates are represented by consecutive dwell-times ($\tau _i$). The position of circles represent the average sequence of dwell-times forming a given cluster. The size of circles are proportional to the normalized cardinality

The phase space representations of the discernible sets of the cross-correlated O–C–O and C–O–C sequences at different channel modulator concentrations (naringenin and quercetin) are presented in Fig. 3. It turns out that the obtained sets of correlated dwell-time sequences form well-distinguishable clusters that give evidence for repetitive patterns in gating dynamics regardless of mitoBK channel stimulation by flavonoids (Fig. 3). The figures characterize the number and spatial organization of identified clusters. In the case of both types of sequences (C–O–C and O–C–O), one can identify 6 well-separated clusters, which are surrounded by the dashed line in Fig. 3. In the case of the C–O–C sequences, one dominating mostly occupied cluster can be indicated (>45% occupancy; C–O–C Cluster I in Fig. 3 (bottom)). In turn, in the case of the O–C–O sequences, two most frequently occupied clusters are evident (>33% occupancy; O–C–O Cluster IV and VI in Fig. 3 (top)). All coordinates of clusters, their cardinalities and their component translations in relation to the appropriate control data are included in Appendices A and B.

A brief visual inspection allows us to observe that the clusters undergo a translation in phase space at flavonoid activation, but, at the same time, changes in cluster occupancy with flavonoid concentration are relatively low (Fig. 3, Appendices A and B). In general, the relative changes in the localization of the centers of the sets of cross-correlated sequences are higher than the changes in their occupancy during mitoBK channel activation by Nar and Que. The average relative difference in the radial coordinate of the sets’ centers obtained at 10 $\mu$M concentration for both flavonoids and the appropriate control data is 29% for the C–O–C clusters and 45% for the O–C–O clusters. In turn, the corresponding average relative changes in clusters’ occupancy are 6% for the C–O–C clusters and 12% for the O–C–Os.

The mitoBK channel activation by naringenin and quercetin does not lead to the change in the number of clusters (that can be related to the constant number of available channel conformations) but affect the relative stability and kinetics of switching between them. These effects seem to be somehow analogous to the BK channel activation by calcium ions, according to the results obtained by Tao and McKinnon by Molecular Dynamics in 2019 (Tao and MacKinnon 2019), where the Ca$^{2+}$ coordination did not affect the number of predefined channel conformations. From this perspective, it can be anticipated that Nar/Que-binding does not lead to notable changes in the tertiary structure of the mitoBK channel protein and, consequently, does not affect the size of conformational space of the mitoBK channel. It may be relatively likely when the most important binding site for channel activation by flavonoids is located rather within the cytoplasmic gating ring than within the transmembrane pore-forming domains.

The literature does not indicate the unequivocal binding sites responsible for the channel activation by flavonoids. In Hsu et al. (2014), the authors analyzed the BK channel activation by naringenin and postulated that it may bind to a site located in the cytoplasmic side of the $\alpha$ subunit (i.e., within the gating ring). This hypothesis seems to be confirmed to some extent by the fact that naringenin exhibits well-pronounced channel-activating effects for different $\alpha$ subunits’ isoforms present in various cell types  (Saponara et al. 2006; Hsu et al. 2014; Yang et al. 2014; Shi et al. 2019; Testai et al. 2013, 2017; Kampa et al. 2019; Kicinska et al. 2020; Da Pozzo et al. 2017), with no or negligible impact of auxiliary regulating $\beta$ and $\gamma$ subunits. Moreover, naringenin coordination exerts discernible effects on gating dynamics from the other stimuli like membrane depolarization (Richter-Laskowska et al. 2022). Thus, Nar-activation seems to exert no effect on the functioning of the voltage-sensing domain (VSD) and the VSD–channel gate interactions.

In turn, according to the docking simulation to the homologous model of the BK channel (based on the chimera of 5TJ6 and 3NAF PDB structures) and the interactions network analysis performed by Fusi et al. (2020), the Thr353 site can exhibit a relatively good affinity for naringenin and quercetin binding. This residue is located in the channel pore. In that study, several minor, but also important, binding sites have been identified, all located close to 353–360 residues. In another study, it has been found that flavonoid quercetin abolishes paxilline inhibition of the mitochondrial BK channel (Kampa et al. 2022). Thus, Que can be coordinated near/by the paxilline binding side inside the channel pore. Like naringenin, quercetin also activates many BK channel isoforms, either the plasma membrane ones (Kim et al. 2011; Zhang et al. 2020; Melnyk et al. 2019) or mitochondrial ones (Kampa et al. 2021, 2022).

From the perspective of the literature mentioned above, the considered flavonoids can be bound to the BK channel in one or more places located within the $\alpha$ subunits, whereas the results of the cross-correlation-based analysis suggest that Nar and Que coordination (regardless of the exact location of the binding site(s)) should not affect sufficiently protein folding to change the number of available channel conformations.

**Fig. 4:**
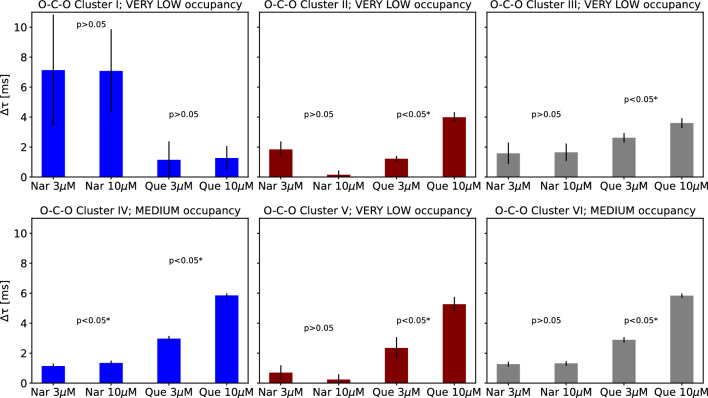
The distances between the centers of the sets of cross-correlated O–C–O sequences obtained at mitoBK channel stimulation by naringenin (Nar) and quercetin (Que) and the corresponding controls. The presented data correspond to the clusters presented and numbered in Fig. 3 (top). Each cluster is tagged by its average occupancy (VERY LOW <10%, LOW 10–35%, MEDIUM >35%). The presented errors on the bar chart are calculated as the standard error of the mean. The statistical significance calculated for $\Delta \tau$s obtained at different concentrations of a given flavonoid (3 and 10 $\mu$M) via non-parametric Mann Whitney *U* test is also included in the graphs. For the statistically significant results at the level of $\alpha = 0.05$, the results are marked with the asterisks

**Fig. 5:**
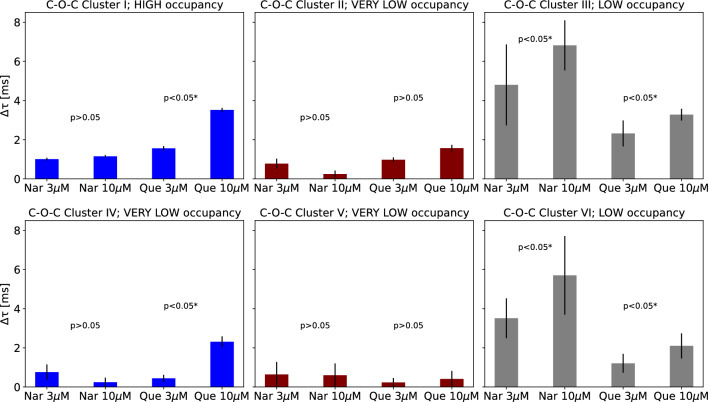
The distances between the centers of the sets of cross-correlated C–O–C sequences obtained at mitoBK channel stimulation by naringenin (Nar) and quercetin (Que) and the corresponding controls. The presented data correspond to the clusters presented and numbered in Fig. 3 (bottom). Each cluster is tagged by its average occupancy (VERY LOW <10%, LOW 10–35%, MEDIUM 35–55%, HIGH >55%). The presented errors on the bar chart are calculated as the standard error of the mean. The statistical significance calculated for $\Delta \tau$s obtained at different concentrations of a given flavonoid (3 and 10 $\mu$M) via non-parametric Mann Whitney *U* test is also included in the graphs. For the statistically significant results at the level of $\alpha = 0.05$, the results are marked with the asterisks

Returning to the results of our analysis, for most clusters, an unidirectional relation between the flavonoid concentration and localization of sets of cross-correlated dwell-time sequences can be observed. The differences in phase space representations between the controls and channels activated by flavonoids are shown in Figs. 4 and 5. Along with the increase of Que concentration, the distance between the centers of O–C–O sets and appropriate controls rises within most of obtained clusters (Fig. 4) and the obtained increment is statistically significant according to the Mann Whitney *U* test (p<0.05). The only exception is exhibited in case of a very low occupied Cluster I, where after the administration of quercetin the occupancy changed by more than $50\%$ (from occ. 16.8% to 4.1%, please see Appendix A). Therefore, in that case one cannot unambiguously formulate a clear interpretation of the direction of spatial changes due to the bias introduced by the “lost” cluster population. There are no unequivocal concentration-dependent effects of Nar-activation on the O–C–O clusters’ localization in most cases, according to the results of statistical tests with the assumed 0.05 p-value.

Greater separation of sets’ centers assigned to different levels of quercetin concentration compared to naringenin (Figs. 4 and 5) can be directly translated into more pronounced effects in case of the mitoBK channel activation by Que than Nar (Table 1), and further, suggests stronger channel interaction with Que than Nar. Consequently, higher binding energy and more detrimental effects for conformational dynamics are anticipated to be exerted by Que than Nar.

Distance between the centers of the Que-related C–O–C clusters and their controls increases with quercetin concentration (Fig. 5) for most of the clusters. Only for two rarely occupied ones (Clusters II and V), the differences were not statistically significant (*p* > 0.05). Structure of the changes of appropriate distances describing the Nar-activation is, however, more complex and not so evident (Fig. 5). It may indicate alternative binding places for the drug with competitive effects.

A more detailed inspection of the changes in O–C–O and C–O–C location induced by channel activation by flavonoids can be performed by comparing the differences within the appropriate coordinates of the dwell-time sequences ($\tau _1$, $\tau _2$, and $\tau _3$). (The appropriate data: $\Delta \tau _1$, $\Delta \tau _2$, $\Delta \tau _3$ are included in Appendices A and B). Effective channel activators are anticipated to prolong the open dwell-times and shorten the closed ones. Basing on the O–C–O data presented in Appendix A, for the most clusters not only open sojourns increased after channel stimulation by flavonoids (of the order of 1 ms) but also the closed ones (of the order of 0.1 ms). The higher effects of the open state prolongation are observed during channel activation by Que than Nar (which is clearly visible in case of the well-occupied clusters IV and VI). Thus, quercetin can be reasonably considered as a better mitoBK channel activator than naringenin. This statement is also confirmed by the corresponding effects of flavonoids on the closed times within the O–C–O dwell-times sequences (Appendix A). The observed increases in closed states’ durations are frequently higher at Nar- than Que-stimulation (please see the results obtained for the popular IV and VI clusters, where the effect of a particular modulator can be attributed to changes in residence time, keeping similar cluster occupancies—the changes $\Delta \tau$ after Que administration can even take negative values compared to positive values obtained after Nar stimulation).

Analogous effects can be observed in case of the C–O–C clusters (Appendix B). For the most frequently occupied cluster (Cluster I), the greater prolongation of the C states is observed at Nar-activation in comparison to Que. On the other hand, the open states are elongated to a higher extent by quercetin than naringenin.

**Fig. 6:**
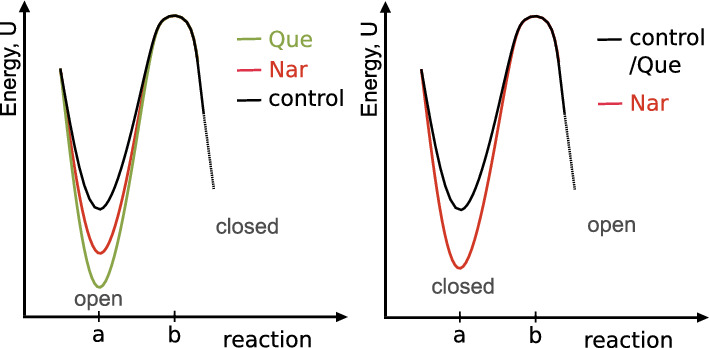
The possible energetic picture of the impact of flavonoid binding (naringenin—Nar and quercetin—Que) on the activation energy ($U(b)-U(a)$) of the conformational change between the most frequently occupied open (O) and closed (C) substates of the mitoBK channel, according to the Kramers Reaction Rate theory. Reaction coordinates “a” and “b” denote the conformation corresponding to the energetic minimum and the potential barrier that separates it from the next state in sequence, respectively. On the left, the energetic changes corresponding to the open-closed switching are depicted. On the right, the changes in energy corresponding to the conformational change from closed to open. Note, the reference potential is unknown—we can infer only about the changes in barrier height

The aforementioned observations can be interpreted in terms of the changes in activation energies of the observed conformational change between the open and closed substates within a considered sequence, as presented in Fig. 6. It seems reasonable to assume that if the lifetime of a given substate is reduced, the potential energy barrier to escape from this substate decreases. In the opposite case, when the lifetime of a substate increases—the potential barrier to escape this state increases. Let us refer to the Kramers Escape Rate formula (Hänggi et al. 1990; Risken 1996):

2 $$\begin{aligned} r=\frac{D}{2\pi k_B T} \sqrt{U^{''}(a)U^{''}(b)}e^{-\frac{U(b)-U(a)}{k_B T}} \end{aligned}$$

where *U*(*x*) is the potential energy landscape along reaction coordinate *x*, with point “a” denoting the considered conformation (substate), and “b” denoting the peak of the potential barrier to escape from state “a”. $k_B$ is a Boltzmann’s constant, T denotes temperature.

One can conclude that the escape rate $r \sim \frac{1}{\tau } \sim e^{-\frac{U(b)-U(a)}{k_B T}}$, neglecting the changes in potential well steepness after binding of channel modulator. This assumption is reasonable if the reaction coordinates “a” and “b”, determining the amount of conformational change, remain separated by a similar distance and the sum of the widths of the potential well and the potential peak remains constant. Consequently, we can obtain the energy barrier value between state *i* and $i+1$ of a sequence as $\tau _i=C_i e^{\frac{U(b)-U(a)}{k_B T} }\rightarrow U(b)-U(a)=k_B T(ln\tau _i - ln C_i)$. In consequence, for the major dwell-time sequences, the activation energies of the corresponding open–closed conformational changes increase in the presence of flavonoids, and the higher energetic barrier separates the mentioned O and C substates in presence of Que than Nar (Fig. 6). In turn, for the most frequently observed closed-open switching, the corresponding changes of dwell-times suggest that the activation energy corresponding to these conformational changes is higher for Nar than Que (Fig. 6). Inferring from the structure of the dwell-time changes corresponding to some low-occupied clusters presented in Appendices A and B, the corresponding changes in the stability of the rarely occupied channel conformations can be more complex and counter-intuitive.

**Fig. 7:**
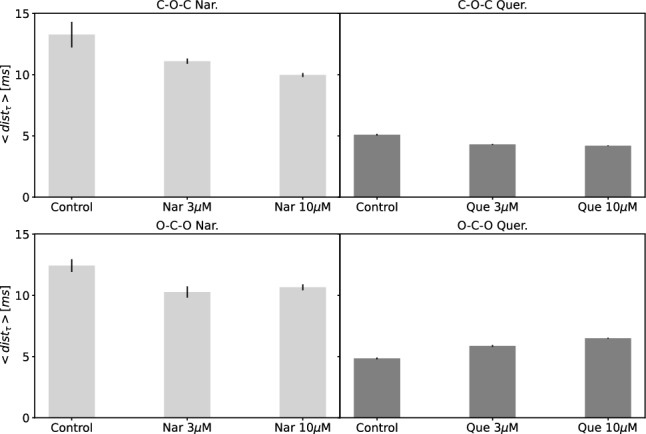
The values of the $<dist_{\tau }>$ parameter for the C–O–C and O–C–O clusters obtained at mitoBK channel stimulation by flavonoids (Nar/Que) and their controls. The presented bar chart errors were estimated by the formula of the total differential

The cross-correlation-based method enables also for observation of the changes in dispersion of the C–O–C and O–C–O clusters in phase space. To evaluate such effects we calculated the <$dist_{\tau }$> parameter for the clusters obtained at fixed external conditions, and the results are presented in Fig. 7. The obtained values of <$dist_{\tau }$> corresponding the O–C–O sequences vary to a similar extent in the case of channel activation by Que and Nar, but the revealed tendencies are opposite. Quercetin binding results in the increase of dispersion of the O–C–O sequences describing gating dynamics, in contrast to coordination of naringenin. The channel modulation by naringenin causes decrease in differentiation in both O–C–O and C–O–C clusters. The aforementioned Nar-related lowering of the dwell-time complexity can be interpreted in terms of the possible decrease in energetic discrepancies within the whole population of open and closed substates of the Nar-bound mitoBK channel (although, for the dominating substates only, opposite changes can be observed.).

The differences in $\Delta p_{op}$, $\Delta \tau$ and $<dist_{\tau }>$ values between the Nar- and Que-activation stem from the different strength of interactions between the flavonoids and the channel. As mentioned before, there can be one or more Nar/Que coordination sites within the $\alpha$ subunits of the BK-type channels. Some of them can be similar for both flavonoids, as suggested in Fusi et al. (2020), where, e.g., the Thr-353 residue of the channel homolog used in molecular docking interacted with the hydroxyl group in the 7 position, present in both Nar and Que, via a hydrogen bond. However, some Nar and Que coordination sites of the channel can be different due to the structural differences between both flavonoids (please see Fig. 1). According to the former results found in literature, these structural differences lead, for example, to the possibility of full-blocking of Nar-activated mitoBK channel by paxilline (at micromolar concentration) (Kampa et al. 2019), while quercetin abolished paxilline inhibition of this channel (Kampa et al. 2022).

The additional observation made in the course of this investigation is the comparison of the effects exerted by different uncontrolled external conditions that can affect the experimental results. The data corresponding to Nar- and Que-stimulation were obtained using different independent mitochondrial isolations from the cells. The cells were cultured according to the same procedure (e.g., at the same temperature, in solutions of the same composition). The patch-clamp recordings were obtained in different experimental days. Thus, mitoplast preparations could be different. Moreover, there could occur additional effects, like changes in atmospheric pressure or cell-to-cell variability. All of these factors could affect the general characteristics of the obtained signals, which can be directly observed in the form of the differences in the results obtained for the controls corresponding to the Nar and Que stimulation (Fig. 3, Appendices A and B). These differences are evident in the case of the clusters of low occupation in contrast to the highly occupied ones, which exhibit generally common characteristics. Since the dominating O–C–O and C–O–C sequences have similar temporal patterns for the control data, one can infer that there exist some generic mitoBK channel stable macroconformations, which are insensitive to some uncontrolled external conditions (mentioned above).

**Fig. 8:**
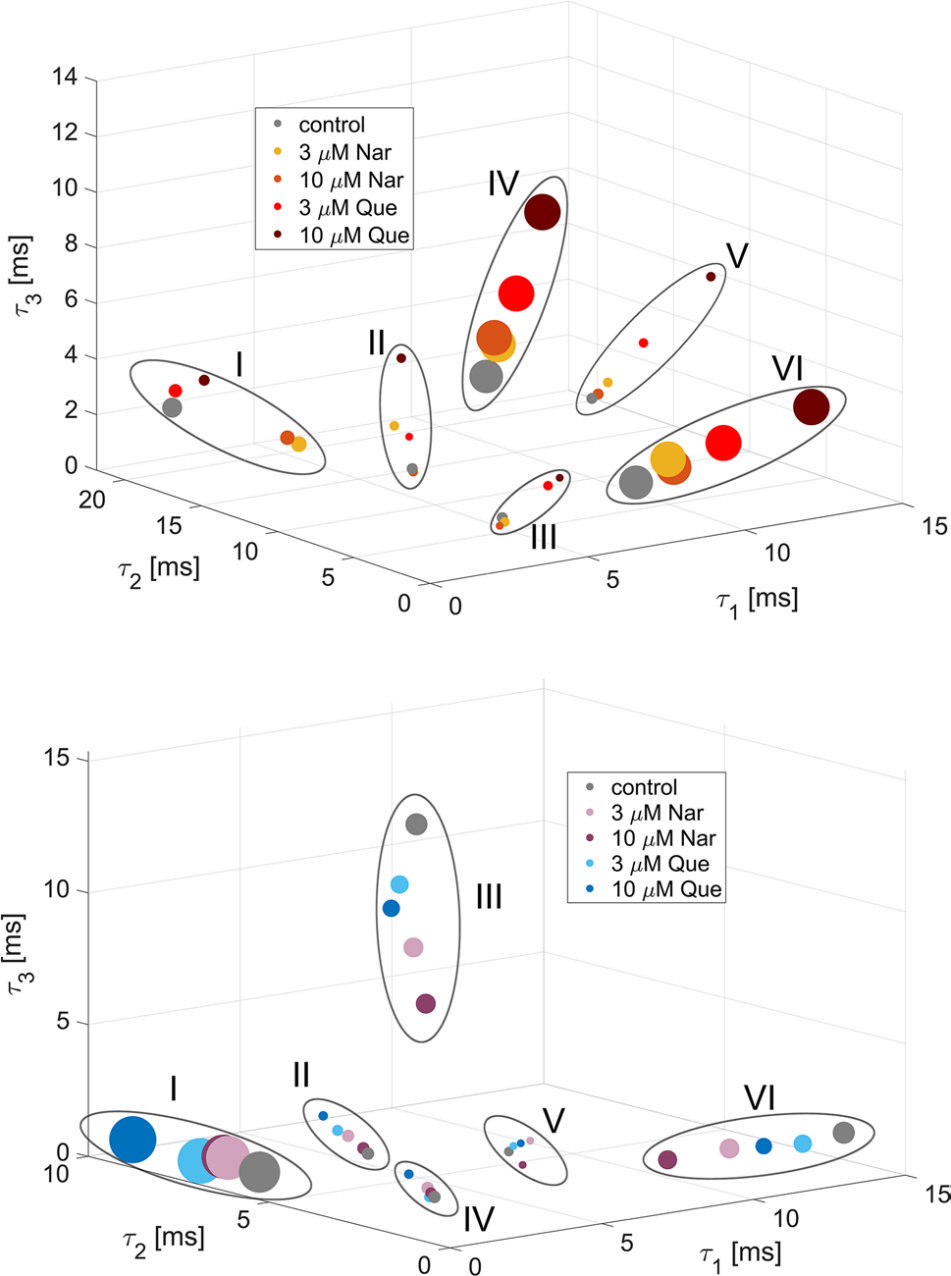
The phase space representation of the clusters of the O–C–O (top) and C–O–C (bottom) sequences describing channel’s conformational dynamics induced by the naringenin and quercetin binding and their common control group. Clusters are encircled and numbered. The coordinates are represented by the dwell-times ($\tau _i$) forming the cross-correlated sequences (at $R_0$ = 0.75) belonging to a given cluster. The size of circles are proportional to the normalized cardinality

Finally, for better visualization of the relative impact of specific modulator concentration on the clusters’ distribution, Fig. 8 characterizes the phase diagrams with the common control group. Owing to such a representation of the obtained O–C–O and C–O–C clusters, the greater effects on the leading sequences exerted by quercetin in comparison to naringenin become evident. Also, the existing unidirectional changes in phase space representation of the mitoBK channel gating caused by the flavonoid stimulation are visible in Fig. 8.

## Conclusion

In this work, we show the applicability of the cross-correlation-based technique (Borys et al. 2022) in the analysis of the effects exerted by the biochemical modulators on the conformational dynamics of the ion channel protein. The methodology gives a possibility of practical monitoring of the changes in temporal characteristics of acts of switching between the functionally different channel conformations in phase space. Thus, it reveals more details about the conformational dynamics of the channel than the basic description restricted to the level of the average macrostate characteristics only (e.g., open state probability, mean dwell-times of open and closed states). We showed here that the properties of the obtained phase portraits in dwell-time space can serve as indicators of how strong a given substance can affect the gating dynamics of a channel.

The effects of naringenin and quercetin binding on conformational dynamics of the mitoBK channel indicated by the correlation-based analysis allowed us to formulate some reasonable hypotheses about the impact of the investigated flavonoids on the number of channel conformations and their relative stability. First, the observed number of clusters does not change with the channel modulation by flavonoids (6 O–C–O and 6 C–O–C clusters in Fig. 3), which suggests that the Nar- and Que coordination to the channel does not affect the number of available channel conformations, i.e., the modulation is rather due to changes in energy barriers than by geometrical blockade of some states.

The increase in concentration of analyzed flavonoids results in better separation of the control data and the ones corresponding to the stimulated mitoBK channels (Figs. 4, 5). In that aspect, the effects of quercetin are higher than naringenin, which allows us to hypothesize that Que-binding causes stronger modification of conformational dynamics (and stronger modification of energetic landscape of conformational space) than Nar-binding. What is, however, important, the values of $\Delta \tau$ differ from cluster to cluster, which suggest that different conformations of the mitoBK channels are affected to a different extent by coordination of flavonoids.

The inspection of the actual changes of open and closed dwell-times within the recognized clusters lead us to an inference, that quercetin is a more effective channel activator than naringenin mostly due to a statistically higher prolongation of open sojourns and weaker effects on closed ones than naringenin (Appendices A and B). It was quite surprising that Nar-binding resulted in stabilization of some closed substates (e.g., O–C–O Clusters 4 and 6 in A). This effect can be considered as another component factor that predetermines naringenin as a weaker mitoBK channel activator in comparison to quercetin. The recognized changes in open and closed dwell-times can be also translated into the description of changes in activation energy needed to escape from open and closed states according to the Kramers Escape Rate theory, as presented in Fig. 6. Quercetin binding results in higher increase of the energetic barrier separating open and closed conformations (corresponding to the most frequently occupied clusters), and this increase can be estimated from Eq. 2 basing on the appropriate open dwell-time durations. Analogously, naringenin binding increases the energetic barrier separating leading closed and open conformations.

The inspection of the dispersion of O–C–O and C–O–C clusters, suggests that Nar-binding reduces the overall dispersion of both types of clusters (that may be a sign of the net lowering of the differences in stability within all available channel conformations) (Fig. 7). In turn, the Que coordination leads to increase of dispersion of the O–C–O clusters, which can be translated to the relatively strong stabilization of some open substates and destabilization of others, in relation to the unbound channel structure. This can be observed also in Appendix A, where the occupancy of the leading clusters (IV and VI) increases with the Que concentration, which is gathered with a relatively large prolongation of the open dwell-times $\Delta \tau _1$ and $\Delta \tau _2$.

## Data Availability

The data that support the findings of this study are available from the corresponding author on request.

## Appendix A: O–C–O clusters: coordinates, translations and occupations

**Table:**

	$\tau _1$	$\tau _2$	$\tau _3$	Occ. %	$\Delta \tau _1$	$\Delta \tau _2$	$\Delta \tau _3$
	Cluster 1						
Control Nar	2.80	32.96	3.03	8.4			
3 $\mu M$ Nar	3.38	25.85	2.93	8.5	0.58	$-$7.11	$-$0.10
10 $\mu M$ Nar	3.03	25.89	3.22	7.3	0.23	$-$7.07	0.19
Control Que	1.09	8.89	1.08	16.8			
3 $\mu M$ Que	1.65	9.84	1.40	6.1	0.56	0.94	0.33
10 $\mu M$ Que	2.03	8.74	1.91	4.1	0.94	$-$0.15	0.83
	Cluster 2						
Control Nar	0.96	3.42	4.26	2.9			
3 $\mu M$ Nar	1.03	4.74	5.54	2.7	0.07	1.32	1.28
10 $\mu M$ Nar	1.02	3.47	4.13	2.8	0.05	0.05	$-$0.13
Control Que	0.69	2.35	2.61	5.7			
3 $\mu M$ Que	0.92	3.03	3.59	2.4	0.23	0.68	0.98
10 $\mu M$ Que	1.18	4.08	6.16	2.8	0.49	1.73	3.56
	Cluster 3						
Control Nar	5.75	5.62	1.04	1.9			
3 $\mu M$ Nar	5.13	4.18	1.27	2.9	$-$0.62	$-$1.43	0.23
10 $\mu M$ Nar	4.96	4.17	1.17	2.2	$-$0.79	$-$1.45	0.13
Control Que	2.55	2.06	0.67	7.0			
3 $\mu M$ Que	4.69	3.49	1.14	3.2	2.15	1.43	0.47
10 $\mu M$ Que	5.41	4.19	1.16	2.0	2.86	2.13	0.49
	Cluster 4						
Control Nar	2.34	0.69	7.51	40.7			
3 $\mu M$ Nar	2.79	0.85	8.55	41.6	0.45	**0.16**	1.03
10 $\mu M$ Nar	2.68	0.87	8.81	41.6	0.34	**0.18**	1.29
Control Que	1.92	0.76	6.29	33.6			
3 $\mu M$ Que	2.84	0.68	9.12	42.7	0.92	**– 0.08**	2.82
10 $\mu M$ Que	3.73	0.80	11.85	43.9	1.81	**0.04**	5.56
	Cluster 5						
Control Nar	5.54	0.57	5.65	4.3			
3 $\mu M$ Nar	6.06	0.60	6.12	2.5	0.52	0.03	0.47
10 $\mu M$ Nar	5.74	0.57	5.76	4.2	0.21	0.01	0.11
Control Que	5.31	0.64	5.36	3.3			
3 $\mu M$ Que	6.95	0.64	7.04	2.7	1.64	0.00	1.68
10 $\mu M$ Que	9.10	0.66	9.01	3.2	3.79	0.02	3.65
	Cluster 6						
Control Nar	7.48	0.68	2.35	41.7			
3 $\mu M$ Nar	8.60	0.87	2.94	41.8	1.11	**0.19**	0.59
10 $\mu M$ Nar	8.77	0.86	2.63	42.0	1.28	**0.18**	0.28
Control Que	6.30	0.77	1.99	33.7			
3 $\mu M$ Que	9.03	0.68	2.91	42.8	2.73	**– 0.09**	0.91
10 $\mu M$ Que	11.90	0.83	3.62	43.8	5.60	**0.06**	1.63

## Appendix B: C–O–C clusters: coordinates, translations and occupations

**Table:**

	$\tau _1$	$\tau _2$	$\tau _3$	Occ. %	$\Delta \tau _1$	$\Delta \tau _2$	$\Delta \tau _3$
	Cluster 1						
Control Nar	0.67	5.92	0.61	65.4			
3 $\mu M$ Nar	0.81	6.90	0.79	63.7	0.14	0.98	0.17
10 $\mu M$ Nar	0.83	7.05	0.78	64.4	0.16	1.13	0.16
Control Que	0.76	5.82	0.80	45.8			
3 $\mu M$ Que	0.68	7.37	0.70	68.6	$-$0.09	1.56	$-$0.10
10 $\mu M$ Que	0.79	9.34	0.80	73.2	0.03	3.52	0.00
	Cluster 2						
Control Nar	0.48	3.19	2.94	3.9			
3 $\mu M$ Nar	0.58	3.83	3.38	4.6	0.11	0.64	0.43
10 $\mu M$ Nar	0.56	3.39	3.06	4.7	0.09	0.20	0.12
Control Que	0.56	2.21	2.15	5.6			
3 $\mu M$ Que	0.40	2.92	2.81	4.1	$-$0.15	0.71	0.66
10 $\mu M$ Que	0.48	3.39	3.19	3.3	$-$0.08	1.18	1.04
	Cluster 3						
Control Nar	0.63	2.25	22.50	12.7			
3 $\mu M$ Nar	0.82	2.49	17.70	13.0	0.19	0.24	$-$4.79
10 $\mu M$ Nar	0.84	2.16	15.68	12.5	0.20	$-$0.09	$-$6.82
Control Que	1.04	1.01	8.23	18.7			
3 $\mu M$ Que	0.69	1.19	5.95	10.9	$-$0.35	0.17	$-$2.28
10 $\mu M$ Que	0.64	1.37	4.99	9.8	$-$0.41	0.36	$-$3.24
	Cluster 4						
Control Nar	3.02	3.05	0.46	4.0			
3 $\mu M$ Nar	3.50	3.64	0.51	4.5	0.48	0.59	0.05
10 $\mu M$ Nar	3.14	3.25	0.52	4.5	0.12	0.20	0.06
Control Que	2.33	2.30	0.57	6.0			
3 $\mu M$ Que	2.57	2.64	0.41	3.9	0.24	0.34	$-$0.16
10 $\mu M$ Que	3.70	4.16	0.53	3.3	1.37	1.86	$-$0.03
	Cluster 5						
Control Nar	3.36	1.06	3.25	0.9			
3$\mu M$ Nar	3.70	0.76	3.70	1.7	0.34	$-$0.31	0.46
10 $\mu M$ Nar	3.23	0.57	2.93	1.9	$-$0.13	$-$0.49	$-$0.31
Control Que	2.50	0.60	2.42	4.3			
3 $\mu M$ Que	2.61	0.56	2.62	1.8	0.11	$-$0.04	0.21
10 $\mu M$ Que	2.75	0.48	2.73	1.7	0.25	$-$0.12	0.31
	Cluster 6						
Control Nar	22.17	2.18	1.15	13.1			
3 $\mu M$ Nar	18.67	2.40	1.12	12.6	$-$3.50	0.22	$-$0.03
10 $\mu M$ Nar	16.47	2.26	1.14	12.0	$-$5.70	0.08	$-$0.01
Control Que	7.56	1.00	1.02	19.6			
3 $\mu M$ Que	6.37	1.14	0.78	10.8	$-$1.19	0.14	$-$0.25
10 $\mu M$ Que	5.55	1.53	0.70	8.7	$-$2.00	0.53	$-$0.32
